# Taking Upper Impressions of the Mouth

**Published:** 1874-05

**Authors:** Geo. S. Fouke

**Affiliations:** Westminster, Md.


### ARTICLE II.

### *Taking Upper Impressions of the Mouth.*

BY GEO. S. FOUKE, WESTMINSTER, MD.

It is admitted that taking impressions of the mouth, particularly of the upper jaw, for dental purposes, is an operation attended with no little uncertainty as to certain and entirely satisfactory results. Therefore, anything tending to simplify and improve the means of performing this very important operation, will not be lightly esteemed by those engaged in supplying artificial dentures.

We propose to consider the practicability of taking upper impressions after a method based upon scientific principles, and which method when rigidly carried out *must* result in a perfectly successful result. Very little has been done as yet to reduce this operation to a strictly scientific basis. It has been hitherto treated as a purely *art operation*, and as such it seems to be regarded as an operation dependent entirely upon the skill and experience of the practitioner.

We believe the operation of taking a practically correct impression for an upper denture, is an operation dependent for its successful performance upon *science* more than upon *art*; and it shall be our object to elucidate the fact in this article.

In considering this operation from a stand-point we have thus assumed, we will first inquire, *what is a practically or philosophically correct upper impression?* Is it the *Plaster-of-Paris* impression which the profession now so generally rely upon as the "perfection," *par excellence* of such impression? We say emphatically, no, it is not.

This *plaster* theory of "perfection" would seem to be self-condemned from this consideration alone, namely, that upper dentures made from the most correctly taken plaster impressions, often result in mere approximations to true suction plates, and sometimes terminate in the most disastrous failures.

Plaster impressions of upper cases are only in the main *ideally* "perfect," and may be, for all that is *known* when taken, a very *incorrect* form of impression for the requirements of the case. Now the reason why the ordinary plaster impression as taken for the upper jaw is deceptive and essentially incorrect in many cases, is simply this: *the uncertainty there is in the proper and requisite distribution of pressure against the membranes of the alveolar ridges and the palatine arch.* The whole secret of this matter of what constitutes a scientifically correct upper impression, lies just in this simple proposition, namely, *distribute* the pressure as it should be in taking the impression, and the impression will be practically correct. Such is our experience.

Now that this matter may be better understood, we will consider a moment. It is said that a perfect upper impression consists in this, to wit: "a correct representation of all the parts to be covered by the plate in their *normal condition.*" Plaster has held a long pre-eminence as the *best*

material for taking impressions, mainly for the reason that *pressure* is avoided in the performance of the operation, and the "parts" are not disturbed in their "*normal condition.*

Fatal error! What an immensity of perplexing trials has resulted from this fallacious theory! We look back upon our own twenty years of experience with *plaster upper impressions* with a sensibly felt shudder! But *science—truth* has lifted the veil, and our burden is lightened.

*Experimentum docet! It* teaches what a practically correct upper impression is, and lays it down as a fundamental principle in the science of taking such impression, that an intelligent distribution of pressure over the surfaces of the ridge and arch of the mouth are an absolute pre-requisite to absolute certainty of practical result. We have exhausted experiment in our own practice, and we feel entirely convinced of the fact, that just in proportion as all the "parts" of the roof of the mouth are perfectly reduced by compression, or intelligently directed manipulative pressure will be the completeness and practical efficiency of the impression. Pressure so applied as to force down all "soft parts" as firmly as possible against the subjacent bones, reduces the entire surfaces to something approximating a uniform, *consistent*, or equally balanced basis or foundation for the resting place of the dental plate. A careful and thorough compression as thus indicated, secures most effectually all that is desirable and necessary in an upper impression. It relieves undue pressure upon the hard *raphæ* of the palatine arch; it sustains the contact of the plate to the "soft" parts; the palatine cavity (so to speak,) is filled by the properly formed plate to its utmost capacity, and as a necessary consequence, the highest degree of usefulness is achieved.

Having endeavored to define what we regard a correct upper impression, we would inquire next *how* is it to be taken in the simplest and most reliable way?

From the nature of the case it is apparent that the ordinary upper impression cups, as made of solid metal, are not adapted to the taking of the kind of impressions we have described. The cups in use, including even Dr. Thomas Wardle's movable palate plate tray, do not afford the operator the means of directing the necessary pressure against the palate. The pressure as made by the metal cups, is made only in one direction, perpendicular to the apex of the arch, and consequently there is great uncertainty in the distribution of the pressure against the soft sides of the palate. With plaster there is simply no pressure at all; and with wax or gutta-percha, the pressure may be but imperfectly made; especially the lateral compression which is so essentially necessary to a correct impression, it is next to impossible to effect.

Finding it simply impossible to carry out our theory with any trays or cups we could find, we devised the upper impression cup for which letters patent were granted us on the 20th of January, 1874.

It is presumed that the profession are aware somewhat of the nature of our new combination impression cup, and it certainly is no part of our design to speak here of its merits for any personal advantage that may accrue to ourself, but solely for the purpose of promoting the interests of the art and science in which we are engaged.

The cup we have devised is a simple, elegant and sure mode of operating. It allows of direct manipulative pressure to be applied to the soft parts of the roof of the mouth after the ordinary perpendicular pressure has been applied. This after manipulative pressure is made against the flexible bottom of the cup directly with the index finger, and in the most certain manner; leaving no possible uncertainty in regard to the intelligent distribution of the requisite pressure. The cup is suited to the use of plaster, wax or gutta-percha, although we confine ourselves almost exclusively to the use of gutta-percha. Wax is very good, and excellent impressions are taken by this process with it.

The mode of manipulating in the use of our cup is readily suggested by the appearance of the tray; but we have some peculiarities in our own use of the cup for the different materials, which we regard as important, and which we propose to give in a future recurrence to this subject.

---

### SELECTED ARTICLES.

---

